# Sternal instability measured with radiostereometric analysis. A study of method feasibility, accuracy and precision

**DOI:** 10.1186/s13019-018-0735-4

**Published:** 2018-05-18

**Authors:** Rikke Falsig Vestergaard, Kjeld Søballe, John Michael Hasenkam, Maiken Stilling

**Affiliations:** 1 Dept. of Cardio-Thoracic Surgery, Aarhus University Hospital, Skejby, Palle Juul-Jensens Boulevard 99, 8200 Aarhus N, Denmark; 2 Dept. of Orthopedic Surgery, Aarhus University Hospital, Tage Hansens Gade 2, 8000 Aarhus C, Denmark; 3 Dept. of Clinical Medicine, Aarhus University, Incuba/Skejby, Bygning 2, Palle Juul-Jensens Boulevard 82, 8200 Aarhus N, Denmark

**Keywords:** Sternum, Wound healing, Bone healing

## Abstract

**Background:**

A small, but unstable, saw-gap may hinder bone-bridging and induce development of painful sternal dehiscence. We propose the use of Radiostereometric Analysis (RSA) for evaluation of sternal instability and present a method validation.

**Methods:**

Four bone analogs (phantoms) were sternotomized and tantalum beads were inserted in each half. The models were reunited with wire cerclage and placed in a radiolucent separation device. Stereoradiographs (*n* = 48) of the phantoms in 3 positions were recorded at 4 imposed separation points. The accuracy and precision was compared statistically and presented as translations along the 3 orthogonal axes. 7 sternotomized patients were evaluated for clinical RSA precision by double-examination stereoradiographs (*n* = 28).

**Results:**

In the phantom study, we found no systematic error (*p* > 0.3) between the three phantom positions, and precision for evaluation of sternal separation was 0.02 mm. Phantom accuracy was mean 0.13 mm (SD 0.25).

In the clinical study, we found a detection limit of 0.42 mm for sternal separation and of 2 mm for anterior-posterior dislocation of the sternal halves for the individual patient.

**Conclusion:**

RSA is a precise and low-dose image modality feasible for clinical evaluation of sternal stability in research.

**Trial registration:**

ClinicalTrials.gov Identifier: NCT02738437, retrospectively registered.

## Background

The median sternotomy has been the preferred way to gain access to mediastinal organs since the dawn of thoracic surgery. The procedure is quick and efficient, and has only two major complications: sternal infection (1–3% of patients) and non-union (2–8% of patients). Sternal non-union is usually a result of primary dehiscence, poor wound healing, or premature overexertion [1].

Some patients have a higher risk of developing sternal instability than others, i.e. patients suffering from morbid obesity, COPD, diabetes mellitus, smoking, and osteoporosis. Few patients are diagnosed with non-union, but up to 56% [2] experience chronic postoperative pain, which might be an indicator of underdiagnosed sternal non-unions.

The clinical diagnosis of sternal instability is determined by manual palpation by a physician and the radiological diagnosis may be confirmed by Computed Tomography (CT). Both methods are correlated with a high degree of intra- and inter-observatory variance. We have previously shown the relative intra-observer variance of radiological evaluation of sternal CT to be a mean − 9.32% (SD ± 16.18), and the relative inter-observer variance to be mean − 14.29% (SD ± 14.88) [3].

We propose the use of a radiostereometric analysis (RSA), which is a low-dose image diagnostic modality, to diagnose sternal instability in clinical studies following cardiac surgery). RSA was developed in 1974 [4] and is today considered the gold standard for evaluation of prosthesis migration in hip and knee arthroplasty [5]. RSA has also been used to assess fracture stability and healing [6]. The safety of RSA is proven through years of use and 99.5% of the beads are stable 6 weeks after implantation [7].

One millimeter radiopaque tantalum beads are used to mark the two fractures pieces. In a pair of x-ray images (stereoradiographs), the three-dimensional bead-positions are reconstructed, resulting in an accurate calculation of the pose of the two fracture pieces. This technique quantifies micromotion with a reported accuracy that ranges between 0.05 and 0.5 mm for translations [8]. Accuracy in this range is certainly sufficient for evaluation of sternal instability [9, 10].

The aim of this study was to investigate the feasibility of RSA in evaluation of the motion between the two sternal halves after median sternotomy and to implement the technique in a clinical population.

## Methods

### Phantom study

Four bone analog phantoms (20pcf density, Sawbones Europe AB, Malmö, Sweden) were subjected to a midline sternotomy. In each sternal half we placed 8 tantalum beads (diameter = 1 mm) by use of a bead-gun (Wennbergs Finmek AB, Gunnilse, Sweden). The sternal halves were reunited using six standard single cerclages. The cerclages were placed to mimic the metal-obscurance seen from wire cerclages in patients. A custom-made separation device for the sternum was built of radiolucent materials, to enable radiographic exposure of the components in all directions (Fig. 2). Images were made with the sternum in 3 different positions to mimic the expected maximal change in motion and angulation of the sternum during a breathing cycle: 1) neutral (sternum perpendicular to the platform), 2) caudal end of sternum elevated to a 15° angle (inspiration), 3) cranial end of sternum elevated to a 15° angle (expiration). The 3 positions were repeated at 4 different separation points (0 mm, 1 mm, 2 mm, 3 mm separation). Twelve radiographs per saw bone were recorded, resulting in 48 stereoradiographs in total.

According to the induced separation of the sternal halves, the difference in means of the measured phantom translations between the 4 separation points should approximate 1 mm in each of the three position groups (neutral, cranial tilt, caudal tilt). We evaluated the accuracy as the measured mean and standard deviation in each of the 3 position groups (neutral, cranial and caudal tilt) in order to show if 1 mm measurements were susceptible to variation in position of the sternum. We further evaluated the translation precision (mm) for each orthogonal axis as the combined mean difference (Mean_diff_) and SD of the mean differences (SD_diff_) of the 3 phantom tilt positions and 4 sternal separations. The Mean_diff_ express systematic errors and the SD_diff_ express the precision of the method. A low SD_diff_ show high precision.

### Clinical study

This study was approved by The Central Denmark Regions Committee on Biomedical Research Ethics as it adheres to the Helsinki Declaration II, and all participants gave their informed consent to participate in this study with the intent to publish.

In the clinical study, approximately 10 tantalum beads (*ø* = 1 mm) were inserted with a bead gun (Wennbergs Finmek AB, Gunnilse, Sweden) in each sternal half during thoracic surgery. 6 weeks after surgery we recorded double-examination stereoradiographs in full inspiration and in full expiration of 7 sternotomized patients (*n* = 28 stereoradiographs). The clinical precision is presented for each orthogonal axis as the coefficient of repeatability (CR) = standard deviation of the mean differences × 1.96. The CR can be perceived as a detection limit for assessment of patient individual migration between th[5]e two sternal halves.

### RSA set-up

**Fig. 1:**
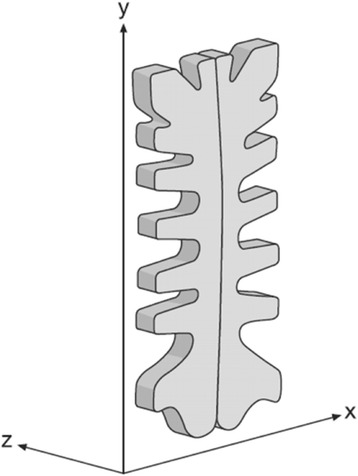
Schematic drawing of axes relating to the anatomy of the sternum

To accurately calculate the positions of the tantalum beads in the saw bones we used a uniplanar focused-grid carbon calibration box (Box 24, Medis Specials, Leiden, the Netherlands) with calibration markers in two layers and film cassettes placed side by side under the calibration box. The calibration box was positioned underneath the x-ray table. The calibration box coordinate system defined the translations (mm) in the frontal plane (the x-axis and y-axis) and in the out-of-plane (the z-axis) (Fig. 1).

A standard RSA setup of two synchronized ceiling-fixed x-ray tubes (Arco-Ceil/Medira; Santax Medico, Aarhus, Denmark), angled toward each other at a 40° angle were used. All stereoradiographs were fully digitalized (FCR Profect CS; Fujifilm, Tvedbæk, Denmark) and were stored without compression. The stereoradiographs that we used for analysis were scaled to 2080 × 2529 pixels (grayscale BMP file-format).

### Analysis of stereoradiographs

**Fig. 2:**
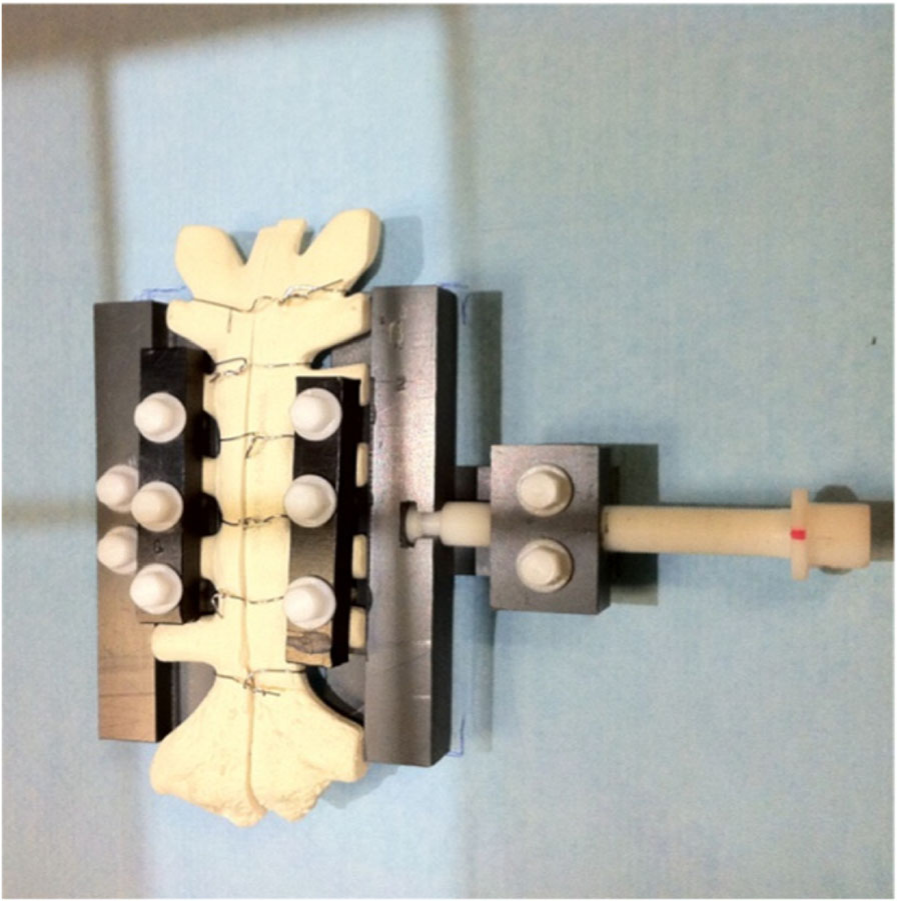
Photograph of the sternal sawbone after median sternotomy and with wire-cerclage placed in the custom-made fixture device

RSA analysis was carried out with model-based RSA 3.31 software (RSA*core*, Leiden, The Netherlands) by one observer (RFV) and the software computed the relative sternal motion. The left sternal half was defined as a rigid body (fixed reference) and the right sternal half was defined as the migrating object (Fig. 2). Migration was measured between the 2 sternal halves with the phantoms in 3 different positions (neutral, and 15° cranial or caudal tilt) along the 3 orthogonal axes (x, y, and z-axis) at each of the 4 separation points (0, 1, 2 and 3 mm separation).

### Statistics

The measured signed translations in the phantom study followed a Gaussian distribution, and therefore statistical comparisons of the means was done with Students paired ttest, and comparison of variations were performed with an f-test. The primary endpoint of the phantom and clinical study was precision of measured migration along the X-axis (frontal plane motion of the sternal halves).

## Results

### Phantom study

We found no systematic error with similar means (*p* > 0.3) between the three phantom positions (Table 1). The combined precision measure of 0.02 mm for assessment of sternal separation (x-axis) in the 3 positions and 4 distractions was very good (Table 1).

**Table 1:** Phantom precision

Phantom study (*n* = 4 phantoms)	X-axis (sternal separation)	Y-axis (proximal-distal translation)	Z-axis (anterior-posterior translation)
Sternum Mean_diff_ (mm)	0.00	0.00	−0.07
SD_diff_ (mm)	0.02	0.04	0.31

The combined mean difference (Mean_diff_) and standard deviation of differences (SD_diff_) for the 3 phantom tilt positions and 4 sternal separations of the 4 phantoms. The Mean_diff_ express systematic errors and the SD_diff_ express the precision of the method

The accuracy for measurement of sternal separation (mean difference for the 3 positions on the x-axis) was − 0.13 mm, since the mean of 0.87 mm approximated the intended 1 mm separation of the jig. There was some underestimation (mean 0.47 mm) for the first separation interval (0–1 mm) and a slight overestimation (0.08 mm) for the two other separation intervals (1–2 mm, 2–3 mm) (Table 2).

**Table 2:** Phantom Accuracy

Phantom Position / Axes	X axis (sternal separation) Mean (SD)	Y axis (proximal-distal translation) Mean (SD)	Z axis (anterior-posterior translation) Mean (SD)
Neutral			
Separation 0–1 mm	0.52 (0.35)	−0.11 (0.16)	−0.53 (0.52)
Separation 1–2 mm	1.05 (0.1)	−0.36 (0.14)	0.04 (0.12)
Separation 2–3 mm	1.06 (0.13)	−0.41 (0.15)	−0.29 (0.22)
*Mean neutral*	0.88 (0.25)	0.29(0.13)	−0.26 (0.23)
15° Cranial tilt			
Separation 0–1 mm	0.54 (0.34)	−0.03 (0.16)	−0.65 (0.32)
Separation 1–2 mm	1.09 (0.08)	−0.35 (0.07)	−0.06 (0.18)
Separation 2–3 mm	1.05 (0.1)	−0.35 (0.23)	−0.44 (0.77)
*Mean 15° cranial tilt*	0.89 (0.25)	−0.24 (0.15)	−0.38 (0.24)
15° Caudal tilt			
Separation 0–1 mm	0.52 (0.31)	0.19 (0.15)	−0.46 (0.43)
Separation 1–2 mm	1.04 (0.07)	0.37 (0.12)	−0.01 (0.16)
Separation 2–3 mm	1.03 (0.11)	0.44 (0.07)	−0.17 (0.37)
*Mean 15° caudal tilt*	0.86 (0.24)	0.33 (0.11)	−0.21 (0.19)

The measured difference in means with standard deviations of an approximated 1 mm x-axis phantom migration on the custom made jig. N = 4

### Clinical study

The mean difference of patient double examination in assessment of sternal separation was 0.07 mm, and the precision (detection limit in individual patients) in terms of the CR was 0.42 mm (Table 3).

**Table 3:** Clinical double examinations

Clinical position / Axes	X axis (sternal separation)	Y axis (proximal-distal translation)	Z axis (anterior-posterior translation)
	Mean (SD)	Mean (SD)	Mean (SD)
*Inspiration (n = 7)*	0.09 (0.24)	− 0.16 (0.30)	0.53 (0.96)
*Expiration (n = 7)*	−0.13 (0.19)	−0.40 (0.36)	1.13 (1.22)
*Combined (n = 14)*	0.07 (0.21)	−0.55 (0.32)	0.67 (1.06)
CR (precision) (*n* = 14)	0.42 mm	0.63 mm	2.08 mm

Double examination results of 14 patient sterni recorded 6 weeks postoperative during inspiration and expiration (28 stereoradiographs). The mean express the change in sternal separation between inspiration and expiration, the SD express the clinical variation, and the CR (coefficient of repeatability = 1.96 x SD_diff_) express the clinical precision of individual RSA for measurements of sternal instability

## Discussion

In clinical research, we should always aim at using the diagnostic tests, which inflict the least danger to the patient, without compromising accuracy and precision, and at a reasonable price. In this study we aimed to investigate the feasibility and validity RSA as a low-dose image modality alternative to CT for evaluation of sternal healing in clinical research.

In the phantom study, we showed the accuracy of the RSA measurements to be acceptable (measurement error of 0.13 mm) with small and statistically non-significant mean differences and variances. Likewise, the precision was at submillimeter level and similar to phantom studies of orthopaedic prostheses [9, 10]. Further, there was no systematic error between the 3 tested sternal positions of the phantom, which simulated the maximal expected clinical change in sternal angulation corresponding to inspiration and expiration.

In the clinical study, double examination stereoradiographs were recorded 6 weeks after surgery where stable tantalum beads in the sternal osteotomy are expected [7]. With repeat stereoradiographs at full inspiration and expiration, we found a clinical precision of less than 0.5 mm for measurement of sternal separation and of 2 mm for measurement of anterior/posterior translation of one sternal half. Dispite cerclage osteosynthesis, up to 2 mm sternal separation has been shown post-operatively at physiological strains [7, 8]. The precision of RSA is certainly sufficient to evaluate sternal instability and non-union at this criteria, as well as to compare the fixation potential of the many different new osteosynthesis techniques currently available to thoracic surgeons. The lower precision in the out-of-plane (Z axis) is a well-known limitation in RSA studies, but it is probably enhanced in our model as a result of tantalum beads being placed almost on a straight line in both the frontal and the sagittal plane [11]. However, this is inevitable because of the flat and narrow anatomy of the sternum.

The radiation dose of 0.8 mSv for RSA is considerably less than the approximate 7 mSv dose for CT. A further advantages of RSA is the lesser economic burden (image recording and analysis) of estimated 46 USD (double examination RSA) compared to approximately 296USD for CT [12]. A limitation of marker-based RSA is the invasive requirement of bead-insertion during surgery; however, the method has proven safe during decades [7]. Furthermore, the radiographic quality of RSA man be lower in patients with high BMI; however, we saw radiographic quality better than the expected standard for thoracic radiographs, and the high atom number of tantalum ensure marker-visibility even with a large soft-tissue bulk. Lastly, the wire-cerclage may occlude the tantalum beads; however, when 10 tantalum beads were inserted per sternum half we did not experience this as a problem for a sufficient marker geometry.

## Conclusion

RSA is a low-dose image modality, which requires insertion of small tantalum beads during surgery and specialized radiographic equipment. The present study confirms the feasibility and precision of RSA for clinical assessment of sternal instability. To the extent of our knowledge, no one has previously used RSA for evaluation of sternal displacement and instability. Currently, RSA is probably the only high-precision, low-dose and safe-proven image modality that can quantify sternal micromotion following median sternotomy as an alternative to conventional CT.

## Abbreviations

CT: Computed Tomography
RSA: Radiostereometric Analysis
